# Cost-benefit analysis of water source improvements through borehole drilling or rehabilitation: an empirical study based on a cluster randomized controlled trial in the Volta Region, Ghana

**DOI:** 10.1080/16549716.2018.1523303

**Published:** 2018-10-01

**Authors:** Seungman Cha, Yinseo Cho, Sharon Jiae Kim, YongJoo Lee, Soonyoung Choi, Patrick Asuming, Yongwhan Kim, Yan Jin

**Affiliations:** a Faculty of Infectious and Tropical Disease, London School of Hygiene & Tropical Medicine, London, UK; b Takemi Program in International Health, Global Health and Population Department, Harvard T.H. Chan School of Public Health, Boston, MA, USA; c Korea International Cooperation Agency, Seongnam, Gyeonggi-do, Republic of Korea; d Independent Consultant, New York, USA; e Team & Team International, Seoul, Republic of Korea; f World Vision Korea, Seoul, Republic of Korea; g Department of Finance, Business School, University of Ghana, Accra, Ghana; h Department of Microbiology, Dongguk University College of Medicine, Gyeongju, Republic of Korea

**Keywords:** Cost-benefit analysis, water source improvements, borehole drilling, borehole rehabilitation, Ghana

## Abstract

**Background**: Despite remarkable progress in water coverage improvements, diseases associated with poor water remain a considerable public health problem in many developing countries.

**Objective**: We aimed to estimate the costs and benefits of drilling or rehabilitating boreholes with handpumps in resource-poor settings and hard-to-reach areas.

**Methods**: Diarrheal reduction in the population was predicted on the basis of the empirical findings from a cluster randomized controlled trial. The full investment and estimated annual running costs were used to calculate the intervention costs. Direct economic benefits of avoiding child diarrheal disease, indirect economic benefits related to health improvements, and non-health benefits related to water improvement were estimated. One-way and multi-way sensitivity analyses were performed to determine the robustness of the findings.

**Results**: Our analysis found that the return on a US$ 1 investment was US$ 9.4 for borehole drilling and US$ 14.1 for borehole rehabilitation. Time savings were the main contributor, accounting for 68% of the benefits, followed by the economic benefits of averted child deaths, which contributed to 15% of the benefits. The sensitivity analyses suggested that improving water sources yields high returns under all circumstances, and that borehole rehabilitation is more efficient than borehole drilling.

**Conclusion**: This study explicitly justifies increased investment in water improvement in rural areas and demonstrates the high returns of rehabilitating boreholes. We hope that this study will be used as evidence for informing the policy decisions of governments or international agencies regarding further investments in improved water coverage in rural areas and the selection of appropriately designed interventions.

## Background

The importance of safe drinking water cannot be overemphasized. The quantity and quality of drinking water in a household play a tremendous role in determining the quality of life of its members, particularly for the people in remote areas in developing countries [1–3]. While the Sustainable Development Goals (SDGs) aim to achieve universal and equitable access to safely managed drinking water for all, 844 million people still lack even basic drinking water services, and many developing countries have therefore prioritized ensuring universal access to at least basic services [4,5]. This ‘unfinished business’ of the Millennium Development Goals (MDGs) – that is, achieving universal access to basic water and sanitation services – requires us to place a particular focus on people in remote, rural, or out-of-reach areas, where 84% of people without basic drinking water live [4]. Unsafe water and poor sanitation cause 1.5 million deaths every year [6,7]. It is well understood that unsafe water has detrimental health outcomes [2,3], but the impact of water goes beyond health. In 8 out of 10 households that do not have water on their premises, women and girls take responsibility for collecting water [4], and thus improving drinking water means improving the quality of life for women and girls. Among the 159 million people who drink surface water, which is classified as unimproved water in the Joint Monitoring Report [4], 58% reside in sub-Saharan African countries, and women and girls may walk for hours to fetch water, even from unprotected sources. In Ghana, only about 81% of the urban population and 51% of the rural population were found to have access to basic drinking water sources [4]. While seeking to ensure universal access to safely managed drinking water, the need to increase resource allocations to basic water services must be continually advocated at international and national levels.

For the purposes of advocacy, the potential impact of improved water is a significant argument for allocating more resources to water service improvement among the various development agendas for poverty alleviation. Unimproved water causes various diseases, including diarrhea, helminthiases, or malnutrition. The World Health Organization (WHO) reported in 2014 that 502,000 deaths were attributed to unsafe and insufficient drinking water [8]. Through ill health, unimproved water can cause death, as well as low educational attainment and delayed cognitive or physical development, leading to low economic productivity [9]. Therefore, improving water could be a way to break this vicious cycle of poverty. As a form of economic evaluation, cost-benefit or cost-effectiveness analysis is the most useful tool for informing resource allocation decisions, especially for government-supported interventions [10–13]. The studies [14–17] done by Hutton and colleagues, and Whittington and colleagues are landmarks in the cost-benefit analysis of water supply and sanitation interventions, but they were carried out on a hypothetical basis. Surprisingly, to our knowledge, few [18–20] economic evaluations have been published on the costs and benefits of improved water and sanitation in rural areas of developing countries, despite the significant amount of investment in this sector. We measured and estimated costs and benefits on an empirical basis using the results of a community-based cluster randomized controlled trial, the results of which have been published elsewhere [21].

Our study compares the costs and benefits of two water source interventions, drilling and rehabilitating boreholes, in the Volta Region of Ghana. Adopting the same methods used in the widely accepted guidelines for cost-benefit analyses of water and sanitation [22,23] published by the WHO, we estimated cost and benefit measures. These included time savings and health outcomes, such as reduction in diarrhea and diarrhea-specific mortality. We aimed to compare the quantifiable benefits gained due to drinking water improvements with the costs of implementing the interventions, including maintenance and operation, expressing both in a common monetary unit. We estimated whether the total benefits of drinking water improvements exceed the total costs of implementation, maintenance, and operation of drilling or rehabilitating boreholes, and investigated the annual rate of return on the investment. In addition, we compared the ratio of returns between drilling and rehabilitation interventions to guide the choice of further water improvement programs that would be most suitable for rural areas. This study also aimed to indicate who would benefit most from these interventions in order to provide relevant information beyond the mere suggestion of economic efficiency.

## Methods

### Study area and intervention

A pair-matched cluster-randomized controlled trial was conducted to explore the effect of source-based water improvements on child diarrhea in the Krachi West and Krachi East districts in the Volta Region, Ghana from March 2012 to December 2014 under the umbrella of the Ghana Volta Region Water, Sanitation and Hygiene project, which was funded by the Korea International Cooperation Agency and implemented by World Vision Ghana. The study area is located 400 km away from Accra, the capital city and the total population is 192,377. Of the 557 communities, 165 were selected for drilling or rehabilitating boreholes, and 78 boreholes were drilled and 83 were rehabilitated. The trial was conducted in 20 communities randomly selected in the two districts. Further details of the intervention, trial design, and evaluation results are described elsewhere [21].

### Data collection and data source

A baseline survey was conducted in October 2012, and the second round of the survey in January 2014 targeted 600 households to explore the effect of improved water sources on child diarrhea based on parental reports. In these two rounds of surveys, we collected caregivers’ monthly income, health facility utilization rate when their youngest under-5 child contracted diarrhea, time and frequency of round trips to collect water for their household, number of diarrhea cases of their youngest child per year, and the person responsible for collecting water. To collect additional data for the cost-benefit analysis, a qualitative study was conducted from December 2016 to January 2017. Four focus group discussions were conducted with two mothers’ groups and two Water, Sanitation, and Hygiene committees in two communities, and students and teachers in four schools. In-depth interviews were conducted in two health centers, two district health management teams, and a district assembly. Information on costs for transportation, food, and drink while visiting health facilities when a child was sick with diarrhea, as well as the consultation fee and prescription charge, was collected during this period. All costs were translated into 2014 values. Secondary data were also collected from the district assembly and World Vision Ghana local offices in the two districts, including the population growth rate and household growth rate, community population, proportion of under-5 children, and proportion of female adults, all of which were reflected when estimating the number of beneficiaries.

### Data analysis

#### Cost measurements

An incremental cost analysis was used, in which all the costs were considered, including the resources to implement and maintain an intervention and other costs resulting from the intervention [24]. We separated the investment and recurrent costs [25]. Hardware, construction, planning, supervision, and education were included in the investment costs, which were drawn from the project records. Maintenance and operations were included in the recurrent costs, which were estimated using the guidelines of the WHO [23]. The operations and maintenance costs were derived using an average of 5% of the annualized costs [23]. For the life span of new boreholes, we referred to the reports of the WHO (average, 20 years; range, 10–30 years) [23]. We estimated the remaining life span of the rehabilitated boreholes as 10 years, since they had been drilled 10 years ago by the Danish International Development Agency (range, 5–15 years) [26].

#### Benefit measurements

##### Time savings

Time savings took place because an improved water source became closer to the households. Time savings helped them to spend more time on leisure or productive work, which imply improvements in well-being or economic value. Daily time savings were drawn from a household survey targeting 600 households. We measured the time for the round trip from each household to an improved source, the frequency of trips to the improved water source per day, and the proportion of adults among those responsible for collecting water. We used the mean value of time saved, representing all the beneficiaries from both borehole drilling and rehabilitation. We did not use different values of time saved by the intervention type because the time saved was not directly associated with whether the borehole was drilled or rehabilitated; instead, it depended on the exact location of the boreholes. All the boreholes were inside each community, meaning that using different values for time saved by intervention type would have generated misleading information on the benefits of drilling or rehabilitating boreholes. When estimating the opportunity costs of time savings [12], we included only adults. To estimate the number of beneficiary adults responsible for fetching water during the borehole lifespan, the population and household growth rate were considered for each community, and we assumed that all community members would benefit from the improved water resulting from the intervention during its life span. The total daily time savings were multiplied by the average hourly income of caregivers, which was also collected from the household survey. To avoid overestimating the intervention, we restricted the opportunity cost to 5 days per week.

##### Health benefits

Reductions in child diarrhea and diarrhea-specific child mortality due to improved water sources were considered when assessing the health benefits. Diarrheal reduction was measured in terms of the prevalence ratio from the community-based cluster randomized trial, and an 11% relative reduction (95% confidence interval: 3%-18%) [21] was found in the intervention group compared with the control group. The effects of water source improvements on child diarrhea found in the cluster randomized trial in the study area are consistent with the results of the latest systematic review [27]. Improved water sources are associated with positive effects regarding pneumonia, nutrition, and numerous other diseases such as helminthiasis, and are obviously beneficial to people of all ages [1], but we restricted the analysis to child diarrhea to derive a sound estimation based only on the data of a robust empirical study, although this led to an underestimation of the beneficial impact.

##### Health sector indirect benefits

In this study, we restricted our analysis of the effects of drilling or rehabilitating boreholes on diarrheal reduction to under-5 children. Since under-5 children are not economically active, we did not translate the reduced morbidity of child diarrhea directly into the opportunity cost of economic productivity. However, averted diarrhea-specific child deaths were translated into opportunity costs [28], since the survival of those children could eventually be linked to economic outcomes. The number of averted deaths resulting from improved water was predicted by multiplying the number of diarrheal cases avoided by the case fatality rate. The diarrheal case fatality rate (lives lost/cases) was estimated at 0.15% [29], which was used to calculate the effectiveness measure (reduction in deaths) for the borehole drilling or rehabilitation program. The convention in traditional cost-benefit analysis is to value deaths avoided at a discounted income stream of the avoided death, from the age at which the person is expected to become productive [28]. The value of time was taken from the survey results. The authors of the above-mentioned study from Peru [28] assumed that members of the population were not economically active when they were over 55. The life expectancy of Ghanaians in 2014 (61.19) was similar to that of Peru in 1993 (66.99). Thus, we also assumed that members of the study population were not economically productive after the age of 55. As in the study from Peru, we assumed that under-5 child deaths occur at an average age of 2 if children die of diarrhea, and if their deaths are averted, the children would not become economically active until the age of 15, meaning that they would have 13 years of no productivity. It was also assumed that they would be economically productive for 40 years, from 15 until 55. The present values of benefits from the averted deaths were calculated after taking into account this 13-year lag period. With this assumption and a 5% discount rate, we derived 9.5 years of discounted productive years lost for under-5 children, using the same method as in previous studies [22,28]. Then, we estimated the income expected to be earned from the averted under-5 child deaths.

##### Health sector direct benefits

We estimated economic benefits in relation to the health care and non-health care costs resulting from fewer cases of diarrhea. Based on the frequency of child diarrheal incidence per year, we calculated the number of diarrheal cases avoided per year among the people who received the intervention. The proportion of caregivers visiting health facilities when their children contracted diarrhea was calculated from the household survey. For the treatment of diarrhea, costs of consultation and treatment were measured by health workers working for the health center. The total savings were calculated by multiplying the unit cost of consultation and treatment by the number of cases averted. Other health-seeking behaviors, such as visiting traditional healers or self-treatment, were excluded due to lack of information, and thus the associated costs were not estimated, which also caused this study to underestimate the actual benefits of the interventions.

##### Non-health sector direct benefits

Transportation costs to health facilities, and other visiting expenses such as food and drink, were estimated as non-health sector direct costs, and were translated into the opportunity costs of time. Caregivers spend more time looking after a child with diarrhea, and additional costs may be imposed due to more intensive care arrangements. In a previous study [14] that investigated who would have been engaging in other productive activities during the time they cared for a sick child, the daily value of the opportunity cost of the child’s caregiver was estimated as 50% of the per capita gross national income per day, suggesting that they would have been engaging in productive activities for 50% of the day if their child had not been sick, and 5 days of opportunity cost were calculated for the benefits. The time savings due to fewer cases of treatment-seeking could be included in the non-health sector direct costs. The duration of illness per case of diarrhea in an under-5 child was estimated to be 5 days in our survey. Taking all these factors into account, we incorporated 2 days of opportunity costs into our estimation instead of 5 days, with the same rationale. To calculate the opportunity cost saved for child care, we multiplied the average hourly income of caregivers, which was collected from the household survey, by 16 hours (2 days) per each diarrheal case prevented.

### Sensitivity analysis

One-way and multi-way sensitivity analyses were performed, with worst and best-case scenarios, to assess the robustness of the estimates and the impact of uncertainty. We analyzed the impacts on different values of key parameters to determine the extent of the reliability of the initial results. Some of the parameters were directly derived from the trial conducted in parallel with the intervention, while the others were adopted from a global perspective [25]. For the life span of boreholes, the discount rate, and the percentage of annualized capital costs for estimating maintenance and operation costs, we referred to the values reported by the WHO [22,23], and for the effects of the water source improvements, we used the results of the trial conducted in parallel with the interventions (Table 1).

**Table 1.** Equations for estimating costs and benefits.

Cost (present value in 2014, discount rate r = 5%)	
Initial investment cost	Total cost of the project invested in drilling and rehabilitating including education
Operation, maintenance, and surveillance	5% annual investment cost^a^
Water source protection	5% annual investment cost
Hygiene education	N/A
Benefit of time savings for collecting water (present value in 2014, discount rate r = 5%)	
Time savings per day (ΔT^W^)^b^	ΔT^W^ = T_0_^w –^ T_1_^w^
Benefit of time savings per day (BTS_d_)^c^	BTS_d_ = ΔT^W^·B_hn_
Benefit of time savings per year (BTS_y_)^c^	BTS_y_ = ΔT^W^·B_hn_·240
Total benefit of time savings (TBTS_a_)^c^	TBTS_a_ =$\sum_{1}^{n}\Delta$ T^W^·B_hn_·P_n_·240·(1 + r)^−n^
Benefit from avoided child diarrhea (present value in 2014, discount rate r = 5%)	
Total benefit of saved transportation from avoided child diarrheal cases (BSTp_a_)	BSTp_a_ =$\sum_{1}^{n}Tp$_n_·ΔD^U5Cn^ ·P_hf_·(1 + r)^−n^
Total benefit of saved food and drinks from avoided child diarrheal cases (BSFD_a_)	BSFD_a_ =$\overset{n}{\underset{1}{\sum }}FD$_n_·ΔD^U5Cn^ ·P_hf_·(1 + r)^−n^
Total benefit of saved consultation cost from avoided child diarrheal cases (BSCo_a_)	BSCo_a_ =$\overset{n}{\underset{1}{\sum }}Co$_n_·ΔD^U5Cn^ ·P_hf_·(1 + r)^−n^
Total benefit of saved treatment cost from avoided child diarrheal cases (BSTr_a_)	BSTr_a_ =$\overset{n}{\underset{1}{\sum }}Tr$_n_·ΔD^U5Cn^ ·P_hf_·(1 + r)^−n^
Total Benefit of time savings for intensive child care from avoided child diarrheal cases (BTSI_a_)	BTSI_a_ =$\overset{n}{\underset{1}{\sum }}16B$_hn_·ΔD^U5Cn^ ·P_hf_·(1 + r)^−n^
Key parameters	
Number of under-5 children in the n^th^ year	U5C_n_
Avoided child diarrheal cases in the n^th^ year(ΔD^U5Cn^)	ΔD^U5Cn^ = (Total number of diarrheal cases)X(relative reduction)
Averted diarrheal-specific child deaths in the n^th^ year (ΔM^U5Cn^)	ΔM^U5Cn^ = ΔD^U5Cn^ X (case fatality rate)
Proportion of caregivers visiting a health facility when their children were sick with diarrhea	P_hf_
Transportation cost for a round trip to the health facility in the n^th^ year	Tp_n_
Cost for food and drinks for the trip to the health facility in the n^th^ year	FD_n_
Consultation cost at the health facility in the n^th^ year	Co_n_
Treatment cost at the health facility in the n^th^ year	Tr_n_
Average days spent caring for the sick child with diarrhea	5 days
Time savings of intensive child care from the avoided child diarrhea per case	16 hours(2 days)
Cost for intensive child care per case	16 B_hn_
aAverage hourly income per caregiver in the project area (US$) in the n^th^ year	B_hn_
Number of adults responsible for collecting water in the n^th^ year after the intervention considering population growth rate, n: life span of boreholes	P_n_

^a^ E = (K-(S/(1 + r)n))/A(n,r) E: the annualized investment cost; K: the purchase price; S: the resale price (assumed to be 0); n: the life span of boreholes; r: the discount rate; A(n,r): the annuity factor; A(n,r) = (1-(1 + r)^−n^)/r (n years at r discount rate). ^b^ T_0_^w^: time for collecting water before the intervention, T_1_^w^: time for collecting water after the intervention (hours).^c^ Assuming that that caregivers could engage in productive work for 240 days per year.

## Results

### Costs and benefits of borehole drilling and rehabilitation

The values of the parameters used to calculate costs and benefits, many of which were measured through the household-based survey, focus group discussions, and in-depth interviews, are presented in Table 2. Improved water sources helped people save 0.6 hours per day, and in 85.8% of households in the community, adults were responsible for collecting water. When translating time savings into opportunity cost, we restricted the analysis to adults fetching water. By virtue of water source improvements, an average of 1.63 diarrheal cases per child were avoided according to the cluster randomized controlled trial. Based on the household survey, the average hourly income of caregivers was US$0.31 and the proportion of caregivers visiting a health center when their children were sick with diarrhea was 72.5%. Based on the case fatality rate of child diarrhea in the previous study, our analysis showed that 217 child deaths would be averted over 20 years in the communities where borehole drilling was performed, while 92 child deaths would be avoided over 10 years in the borehole-rehabilitated communities. Table 2 also summarizes the number of beneficiaries, the number of avoided child diarrheal cases, and the number of averted child deaths both in the first year after the intervention and over the entire life span of the boreholes.

**Table 2.** Values of the parameters for calculating benefits and costs.

Item		Value		Information source
Common values of both groups				
Time saved for fetching water per day per household		36 minutes (0.6 hours)		Household survey
Average number of households per community		287		District assembly strategy
Proportion of households where the age of members responsible for fetching water is 18 or above		85.80%		Household survey
Population growth rate		2.65%		District assembly strategy
Relative reduction of diarrheal prevalence		11%		Cluster randomized controlled trial
Number of diarrheal episodes reduced by borehole drilling or rehabilitation per child per year		1.63 cases		Cluster randomized controlled trial
Proportion of caregivers taking a child with diarrhea to a health facility		72.5%		Household survey
Average time for a round trip to the health facility		8 hours		Household survey
Average income of caregivers per hour		US$ 0.30787		Household survey
Discounted productive years of under-5 children		9.5 years (3.1–16.2 years)		Discount rate: 5% (3%-10%)
Different values by intervention		Borehole drilling	Borehole rehabilitation	Information source
Number of communities		65	59	Project final report
Life span of boreholes		20 years	10 years	WHO, survey
The first year after the intervention (2014)	Number of beneficiaries	40,740	39,745	Project final report
	Number of adults responsible for collecting water	4749	4633	Survey
	Number of under-5 children	3422	3339	Project final report
	Number of child diarrheal cases avoided	5578	5442	Survey
	Number of child deaths averted	5	5	Estimation
Accumulated (2033 for borehole drilling; 2022 for rehabilitation)	Number of beneficiaries	1,056,543	448,356	Estimation
	Number of adults responsible for collecting water	123,168	60,918	
	Number of under-5 children	88,750	37,662	
	Number of child diarrheal cases avoided	144,662	61,389	
	Number of child deaths averted	217	92	

**Figure 1.**
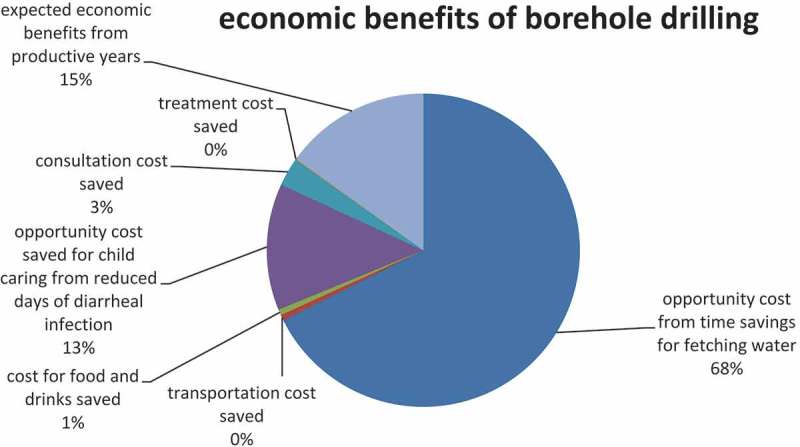
Proportion of benefits by item (in borehole drilling communities).

The results for the benefits and costs are presented in Table 3. The costs for operation, maintenance, surveillance, and water source protection were derived using an average of 5% of the annualized investment cost. The total cost was estimated at US$ 948,376 in the communities where boreholes were drilled and US$ 281,241 in the communities whose boreholes were rehabilitated. The total economic benefit was estimated to be US$ 8,883,325 for 20 years in the beneficiary communities where boreholes were drilled and US$ 3,976,432 for 10 years in the communities where boreholes were rehabilitated. The cost-benefit ratio was 9.4 for borehole drilling and 14.1 for borehole rehabilitation, suggesting that the borehole rehabilitation program was more cost-beneficial than the borehole drilling program in this empirical cost-benefit analysis study. Figure 1 shows that time savings were the main contributor, accounting for 68% of the benefits followed by the economic benefits of averted child deaths, which contributed to 15% of the benefits.

**Table 3.** Economic benefits and costs of borehole drilling and rehabilitation (present value in 2014).

		Communities of borehole drilling	Communities of borehole rehabilitation
Benefit	Opportunity cost from time savings for fetching water	6,006,466	2,548,910
	Transportation cost saved	52,440	22,253
	Cost for food and drinks saved	52,440	52,440
	Opportunity cost saved for child care from reduced days of diarrheal infection	1,161,526	492,906
	Consultation cost saved	262,200	111,267
	Treatment cost saved	7866	3338
	Expected economic benefits from productive years	1,340,387	318,532
Cost	Initial investment on borehole drillings	817,990	234,308
	Maintenance and operation	65,193	23,467
	Water quality control	65,193	23,467
	Total benefit	8,883,325	3,976,432
	Total cost	948,376	281,241
Benefit-cost ratio		9.4	14.1

### Sensitivity analysis

**Figure 2.**
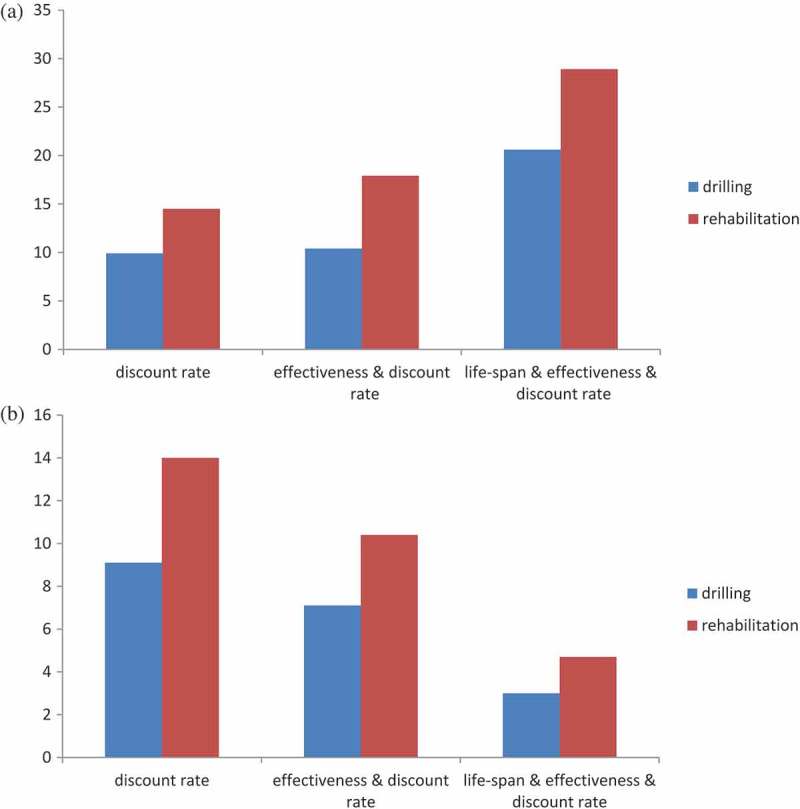
(a) Cost-benefit ratio(y-axis) in best case scenario. (b) Cost-benefit ratio(y-axis) in worst case scenario.

One-way and multi-way sensitivity analyses were used to assess the extent to which variations in the assumptions regarding the various parameters would affect the cost-benefit ratio, as presented in Table 4. Figure 2 suggests that borehole rehabilitation had higher cost-benefit ratio both in the worst- and the best-case scenarios. The results of the sensitivity analysis reaffirm that the initial results of the cost-benefit analysis were reliable, and borehole rehabilitation interventions were more favorable in all circumstances.

**Table 4.** One-way and multi-way analysis of the cost-benefit ratio.

	Borehole drillings			Borehole rehabilitation		
	Base case	Worst case	Best case	Base case	Worst case	Best case
Sensitivity on discount rate						
Discount rate	5%	10%	3%	5%	10%	3%
Total cost	948,376	878,013	997,652	281,241	266,977	288,822
Value of avoided mortality	1,340,387	437,389	2,285,712	318,532	103,942	543,182
Total benefits	8,883,325	7,980,327	9,828,650	3,976,432	3,761,842	4,201,082
Net present value	7,934,949	7,102,314	8,830,998	3,695,191	3,494,865	3,912,260
Benefit-cost ratio	**9.4**	**9.1**	**9.9**	**14.1**	**14.0**	**14.5**
Sensitivity on effectiveness & discount rate						
Discount rate	5%	10%	3%	5%	10%	3%
Effectiveness of intervention	11%	3%	18%	11%	3%	18%
Total cost	948,376	878,013	997,652	281,241	266,977	288,822
Value of avoided mortality	1,340,387	119,288	3,740,256	318,532	50,621	1,587,219
Total benefits	8,883,325	6,232,544	10,387,463	3,976,432	2,772,749	5,175,434
Net present value	7,934,949	5,354,531	9,389,811	3,695,191	2,505,772	4,886,612
Benefit-cost ratio	**9.4**	**7.1**	**10.4**	**14.1**	**10.4**	**17.9**
Sensitivity on life-span & effectiveness & discount rate						
Discount rate	5%	10%	3%	5%	10%	3%
Effectiveness of intervention	11%	3%	18%	11%	3%	18%
life span	20 years	10 years	30 years	10 years	5 years	15 years
Total cost	948,376	932,041	970,946	281,241	272,972	288,474
Value of avoided mortality	1,340,387	51,888	6,485,320	224,650	23,658	2,550,762
Value of time savings	6,006,466	2,612,710	10,414,757	2,548,910	1,191,240	4,096,262
Value of avoided morbidity	1,536,472	128,915	3,085,705	1,202,872	78,506	1,687,005
Total benefits	8,883,325	2,793,513	19,985,782	3,976,432	1,293,404	8,334,029
Net present value	7,934,949	1,861,472	19,014,836	3,695,191	1,020,432	8,045,555
Benefit-cost ratio	**9.4**	**3.0**	**20.6**	**14.1**	**4.7**	**28.9**

## Discussion

This study explored the cost-benefit ratio of water source improvement interventions against the backdrop of growing demand for information regarding the development-wide effects of improved accessibility of safe drinking water. The collective costs and community-wide economic returns of borehole drilling and rehabilitation interventions were estimated on an empirical basis, using reliable evidence from a cluster randomized controlled trial. This empirically based analysis showed that despite the use of conservative assumptions, communities received an average of 14-fold and 9-fold economic returns from borehole rehabilitation and borehole drilling, respectively. This cost-benefit ratio is remarkable compared with other health interventions frequently cited as highly effective and beneficial, such as micronutrient fortification or mass drug treatment for children [17]. This study underscores that improvements in drinking water are worthy of investment and must continue to be prioritized among diverse agenda items in low- and middle-income countries.

Another key finding of this study is that borehole rehabilitation yielded higher-economic returns than borehole drilling under all circumstances. Boreholes are the most common type of water source in rural areas of sub-Saharan African countries. In sub-Saharan Africa, about 60,000 new handpumps are installed every year, but around one-third of rural boreholes with a handpump have been estimated to be nonfunctional [30,31]. One of the key reasons was reported to be insufficient attention to operation and maintenance [30]. During the MDG campaign period, the water component of the MDG targets was achieved in 2010. However, since a substantial percentage of water facilities were reported to be malfunctioning [31], improved water coverage should be continually monitored. Attention should be drawn to the high returns of rehabilitating boreholes, since the SDGs emphasize a continuous supply of improved water.

The global community should take immediate action to rehabilitate broken boreholes, including the development of sustainable measures to help communities fully utilize them for the entire lifespan of the improved sources.

The main limitation of this study lies in its underestimation of the effect due to the strict application of an empirical basis. Previous studies [15,16] estimating economic returns on a theoretical basis took into account avoided morbidity in all age groups, categorized into adults, school-aged children (5–15 years of age) and under-5 children. For adults, two days were assumed to be gained per each diarrheal case avoided, and the per capita gross national income was used as the value of time. School aged children were assumed to miss school for three days, and the same value of per capita gross national income was used as for adults to estimate the social and economic implications of children missing school on development [32]. We restricted the avoided-morbidity effects of water improvements to under-5 children in order to ensure that our estimates utilized the empirical evidence collected in the intervention area. Considering the biological plausibility of its disruptive role in the fecal-oral transmission cycle, the economic returns of both borehole drilling and rehabilitation in this study were underestimated, since improved water would also bring about health benefits to all the other age groups. In addition, we did not calculate a range of indirect benefits of water improvements such as impacts on child nutrition, educational performance, helminthiasis, and pneumonia because we sought to apply only empirical evidence from the study area, although robust evidence exists for causal relationships between water improvement and these outcomes [9]. Therefore, caution is needed when comparing our findings with the cost-benefit ratios reported for other health interventions. Nonetheless, it is worth noting that water improvements brought remarkably high returns on investment to community members.

Hutton [15] indicated that the benefit-cost ratio was most sensitive to the value of time. In our study, the value of time was collected from a household survey, which was subject to measurement errors, because most of the community members were farmers and/or petty traders (e.g. selling agricultural produce at traditional markets or on the street), and the monthly income they reported might have been imprecise. The hourly income in this study, US$ 0.31, was 42% of the hourly gross domestic product (GDP) per capita of Ghana in 2014. Considering that previous studies used 30% or 100% of hourly GDP per capita, the value of time used in our study seems to be in an appropriate range.

The global costs and benefits analysis [15] indicated that mortality reduction contributed to 28% of the total benefits in the sub-Saharan Africa region. The somewhat larger share of the contribution from mortality reduction in the global analysis [15] appears to have resulted because the researchers included averted cases of various diseases apart from diarrhea, such as helminthiasis and malnutrition-related diseases, and also included the effects of water improvement on all age groups, not only under-5 children.

The benefit-cost ratios in previous studies [14,15] were smaller than those found in this study, although they included more various indirect effects. For example, Hutton and colleagues estimated the benefit-cost ratio to be 2.0 for the world and 2.5 for the sub-Saharan Africa region. They considered the lifespan of a borehole to be 30 years, whereas it was 20 years in our study. Apart from this, the values of many parameters in our study, including the value of time and the amount of time savings, were more conservative. We included all the cost elements incurred as part of implementing the project, not only the direct price of borehole drilling or rehabilitation, and we strictly applied the methods of the WHO for calculating the cost of maintenance and operation. The initial investment cost was US$ 10,487 for drilling one borehole and US$ 2,823 for rehabilitating one nonfunctioning borehole (model: Indian Mark II or Afridev). The investment cost for drilling a borehole seems to be reasonable based on similar projects undertaken in sub-Saharan countries [33]. The higher benefit-cost ratios might be attributed in part to the larger number of beneficiaries. The number of beneficiaries per borehole was an average of 499 persons in this empirical study, although the recommended maximum number is 300. Therefore, caution is needed when interpreting the results because the high pressure caused by a greater number of users may cause boreholes to have shorter lifespan. However, considering that this analysis did not include the health effects of the intervention on people aged above 5, other health effects apart from diarrhea, or educational effects, all of which were included in previous studies [14,15], the results of this study seemed to be unlikely to overestimate the benefit-cost ratio.

The benefits and costs of repairing boreholes vary depending on the reason for nonfunctioning and their remaining lifetime. In many cases, boreholes with handpumps could be fixed at less cost than was estimated in this study [34], and many of the water sources in rural areas of Ghana were found to have broken down much earlier than anticipated (3–10 years after installation) [35], suggesting that the benefit-cost ratio of rehabilitating boreholes could be higher than the values estimated in this study. All in all, we believe that our study indicates that investment in water improvements in rural areas has a substantial benefit, regardless of whether boreholes are drilled or rehabilitated.

## Conclusions

In order to achieve universal coverage of safe drinking water in developing countries, economic arguments regarding the high returns from borehole drilling and rehabilitation are critical because such arguments can be used to support greater resource allocation in this field. This study explicitly justifies increased investment in water improvements in rural areas and demonstrates the high returns of rehabilitating boreholes. We hope that this study will be used as evidence for informing the policy decisions of governments or international agencies regarding further investment in improved water coverage in rural areas and the selection of appropriately designed interventions, although we recognize that further evidence is still needed in order to capture the indirect effects of drinking water improvement.

## Paper context

Cost-benefit and cost-effectiveness analysis are used in economic evaluations to inform resource allocation decisions. Studies on the costs and benefits of water improvements are typically based on global or regional estimates which may not apply at the local level. Economic evaluations that take contextual factors into account are needed to inform decisions about water improvements in low-resourced settings. This study uses local data from a cluster randomized controlled trial to assess the costs and benefits of drilling or rehabilitating boreholes with handpumps in Ghana. To our knowledge, this is the first empirical study of its kind. The findings show that water improvements in rural areas yield high economic returns, and that borehole rehabilitation is more efficient than borehole drilling.
